# Preoperative Body Composition Correlates with Postoperative Muscle Volume and Degeneration after Total Hip Arthroplasty

**DOI:** 10.3390/nu16030386

**Published:** 2024-01-29

**Authors:** Taku Ukai, Katsuya Yokoyama, Masahiko Watanabe

**Affiliations:** 1 Department of Orthopaedic Surgery, Surgical Science, Tokai University School of Medicine, 143 Shimokasuya, Isehara 259-1193, Japan; 2 Department of Orthopaedic Surgery, Tokai University School of Medicine Oiso Hospital, 21-1 Gekkyo, Oiso 259-0198, Japan

**Keywords:** bioelectrical impedance, body composition, muscle mass, total hip arthroplasty

## Abstract

Impaired muscle recovery after total hip arthroplasty (THA) may affect gait and activities of daily living. Bioelectrical impedance analysis (BIA) can assess body composition and muscle volume, and computed tomography (CT) can assess muscle volume and the fatty degeneration of muscle. This study aimed to explore the effectiveness of BIA, and the correlation between preoperative body composition and postoperative muscle volume and degeneration after THA using BIA and CT. Thirty-eight patients who underwent THA and had BIA and CT performed pre- and postoperatively were retrospectively assessed. The BIA-derived measurements of preoperative body composition (fat mass index, fat-free mass index, and phase angle) were correlated with the CT-derived measurements (pre- and postoperative muscle volume and gluteus maximus and quadriceps Hounsfield Units of the affected hip). The preoperative fat mass index negatively correlated with the postoperative muscle volume of the gluteus maximus (*p* = 0.02) and quadriceps (*p* < 0.001) and the Hounsfield Units of the gluteus maximus (*p* = 0.03) and quadriceps (*p* = 0.03). The preoperative fat-free mass index positively correlated with the postoperative muscle volume of the quadriceps (*p* = 0.02). The preoperative phase angle positively correlated with the postoperative muscle volume of the quadriceps (*p* = 0.001) and the Hounsfield Units of the gluteus maximus (*p* = 0.03) and quadriceps (*p* = 0.001). In patients who underwent THA, preoperative body composition correlated with postoperative muscle volume and the fatty degeneration of the affected lower limb. Preoperative body composition may help predict postoperative muscle volume and fatty degeneration and thus, postoperative recovery.

## 1. Introduction

As population aging has progressed, the number of total hip arthroplasties (THAs) performed has been increasing. Globally, the number of THAs is expected to increase by 174% from 2005 to 2030 [1]. THA reduces hip pain and increases quality of life [2]. Although THA enables patients who suffer from hip pain to regain an almost normal gait balance [2,3], this balance is affected by the strength of the individual muscles of the hip [4,5]. Muscle mass, strength, and physical function greatly vary among individuals [6]; moreover, muscle volume is associated with the daily life function, internal medical diseases, and quality of life. Muscle volume not only strongly correlates with joint torque [7,8], but also affects common diseases such as cardiovascular disease and diabetes [9,10,11]. Spino-pelvic and spino-femoral muscle volume is related to spinal deformity that causes disability of active daily living [12,13]. Manabe et al. described the association of the psoas muscle volume with sarcopenia, which worsens the quality of life [14]. Furthermore, postoperative muscle strength affects THA outcomes [15,16]. Nankaku et al. reported that muscle strength affected ambulatory ability six months after THA [15], while Holstege et al. demonstrated that greater quadriceps strength was associated with better clinical outcomes after THA [16]. Some authors have reported that muscle volume increased and muscle atrophy was attenuated after THA [17,18], and others showed that muscle volume was related to muscle strength [19]. These reports support the importance of predicting muscle volume after THA.

Computed tomography (CT) is used to evaluate both muscle volume and muscle degeneration using Hounsfield Units (HUs) [17,20,21,22]. Different body tissues can be distinguished based on their respective densities, such as fat (−100 HU), water (0 HU), muscle (30–50 HU), and bone (400–1000 HU) [23]. Therefore, the fatty degeneration of muscle after THA may be assessed using HUs and CT [18,24]. Although CT is helpful for evaluating muscle volume and fatty degeneration, radiation exposure is a concern, and less invasive devices that can assess muscle volume and adipose degeneration would be preferable. Body composition parameters, such as bone mineral content and body fat, have recently gained attention because of their potential association with physical function [25]. Therefore, a simple and non-invasive apparatus would be helpful to assess body composition. Bioelectrical impedance analysis (BIA) is widely used for assessing an individual’s clinical condition [26] and obesity [27]. By using a harmless electrical current, BIA can simply and non-invasively assess the fat mass index (FMI) by using the following formula: fat mass (kg)/[height (m)^2^] and the fat-free mass index (FFMI) by calculating free fat mass (kg)/[height (m)^2^] [28,29]. Additionally, BIA is widely used to assess segmental skeletal muscle mass [30,31]. Skeletal muscle volume directly affects sarcopenia, and decreased muscle strength may cause a limping gait after THA; thus, an accurate assessment of skeletal muscle is important. BIA can also assess the phase angle (PhA), which represents the angle between electrical impedance and cell membrane resistance [32]. The PhA is also known to be associated with cellular health, body cell mass, cell membrane integrity [32,33,34], muscle mass [35], body fat [36], and malnutrition [37,38], in addition to aiding the improvement of muscle quality [39], muscle strength [40], and functional status [41,42].

Although preoperative body composition can affect muscle volume and degeneration after THA, to the best of our knowledge, no previous study has elucidated the correlation between preoperative body composition and muscle volume and adipose degeneration after THA. Therefore, the purpose of this study was to determine whether preoperative body composition (FMI, FFMI, PhA) correlates with muscle volume and/or muscle adipose degeneration after THA.

## 2. Materials and Methods

The required sample size was estimated using G power version 3.1.9.2, assuming the following parameters: the *t*-test analysis between correlation (point biserial model), a moderate effect size (d = 0.5), an alpha error of 0.05, and 80% power. The required sample size was 26 participants. This study retrospectively investigated 38 patients who underwent THA between 2018 and 2021. Demographic data are shown in Table 1. All procedures were approved by the Institutional Review Board of the University of Tokai (21R-311). Participants underwent both BIA and CT one month before and six months after THA. The affected side muscles were evaluated pre- and postoperatively. The inclusion criteria were as follows: (1) patients who underwent BIA and CT pre- and post-THA and (2) patients who could walk without a walker before THA. The exclusion criteria were as follows: (1) patients who used a wheelchair before THA and (2) patients who had undergone ipsilateral surgery around the hip.

All patients underwent THA using the anterolateral supine approach. The same acetabular components (Continuum; Zimmer Biomet, Warsaw, IN, USA) and femoral components (Fitmore; Zimmer Biomet, Winterthur, Switzerland) were placed in all patients. Full weight-bearing was allowed from the day after the surgery. Rehabilitation time and frequency were unified.

Muscle strength was measured both one month before and six months after surgery using a handheld dynamometer (Anima, μ-TasF1, Tokyo, Japan). Hip flexion and abduction were measured in a supine position. Hip extension was measured in a standing position. Measured muscle strength was standardized by body weight (N/kg).

Height was measured to the nearest 0.1 cm using a fixed stadiometer. Body weight was measured using a digital scale (seca, Hamburg, Germany) to the nearest 0.1 kg in the standing position. The body mass index (BMI) was calculated as body weight (kg) divided by height, squared (m^2^).

**Figure 1:**
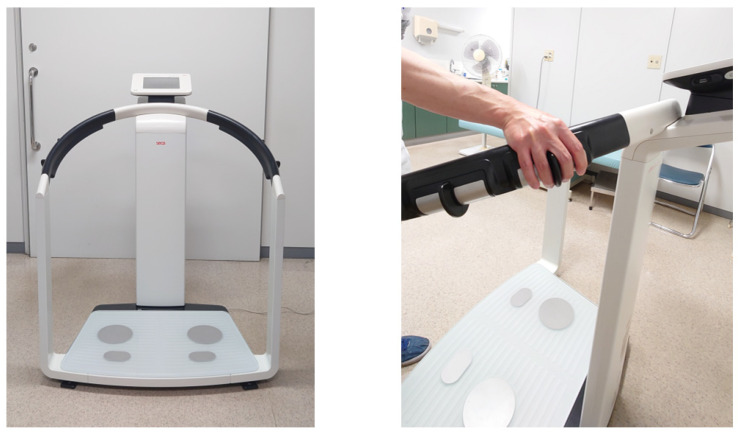
A bioelectrical impedance analysis device for body composition measurements. Bioelectrical impedance analysis takes advantage of noninvasive minute electric current (100 µA) using two pairs of hand electrodes and two pairs of foot electrodes. This device (mBCA515; seca, Hamburg, Germany) can measure segmental skeletal muscle mass (right arm, left arm, trunk, right leg, left leg), fat mass index, fat-free mass index, and phase angle simultaneously.

Body composition (FMI, FFMI, and PhA) was measured using multi-frequency BIA (mBCA515; seca, Hamburg, Germany, Figure 1). This device can also separately measure impedance in the limbs at 19 frequencies ranging from 1 to 1000 kHz. The FMI, FFMI, and PhA were calculated from the manufacturer’s proprietary software. Participants’ body composition was measured once in the standing position, with four electrodes in contact with the feet and four with the hands. Participants were instructed to remain in the standing position during the scan period of 17 s. To minimize the variations in the body water balance, BIA was performed under the same temperature and humidity conditions. Patients were instructed to refrain from eating and drinking before the BIA procedure.

**Figure 2:**
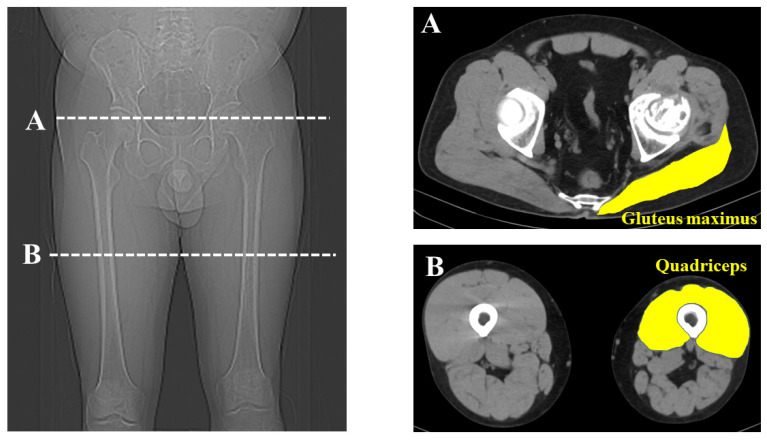
Measurement of muscle volume and Hounsfield Units. Cross-sectional measurement of the gluteal maximus and quadriceps was performed using computed tomography images at the greater trochanter (**A**) and the middle of the thigh (**B**).

CT was performed from the range of the anterior superior iliac spine (ASIS) to the knee joint (Siemens, Germany; 120 kV, slice thickness; 0.6 mm, 0.5–1 s scan time). The anterior pelvic plane was defined using landmarks of the bilateral ASIS plane and pubic tubercles [17]. The cross-sectional area (CSA) of the gluteus maximus of the affected side was measured at the most proximal point of the affected greater trochanter. The CSA of the affected quadriceps was measured at the middle of the affected thigh. Considering each participant’s physique, the measured muscle volume was normalized for each patient’s body weight (mm^2^/kg). Each muscle was measured by manual tracing and HUs were derived using imaging analysis software DICOM (syngo.via version VB60) in a 512 × 512 pixel format (Figure 2). Two observers separately measured each muscle twice and the average value was used for evaluation.

The Shapiro–Wilk test was used to identify the normality of the data and revealed that the preoperative HU values of the gluteus maximus, postoperative HU values of the quadriceps, and postoperative muscle volume of the gluteus maximus and quadriceps did not conform to a normal distribution. Thus, non-parametric statistical analyses were used. The Wilcoxon signed-rank test was used for evaluating the pre- and postoperative muscle strength, FMI, FFMI, PhA, muscle volume, and HU of the gluteus maximus and quadriceps. Spearman’s rank correlation test was performed to assess the correlation of the preoperative FMI, FFMI, and PhA and postoperative muscle volume and HUs. All tests were performed at a significance level of *p* < 0.05. Analyses were performed using SPSS statistical software version 26 (IBM Corp., Armonk, NY, USA).

## 3. Results

Regarding pre- and postoperative parameters, postoperative hip flexion, extension, and abduction, muscle strength was significantly higher than the preoperative values (*p* < 0.01). The postoperative FFMI was significantly higher than the preoperative FFMI (*p* < 0.01). The postoperative muscle volume of the gluteus maximus and quadriceps was significantly higher than the respective preoperative volumes (both *p* < 0.01). No significant difference was seen between the pre- and postoperative FMI, PhA, and HUs, both of the gluteus maximus and quadriceps. These data are recorded in Table 2.

**Figure 3:**
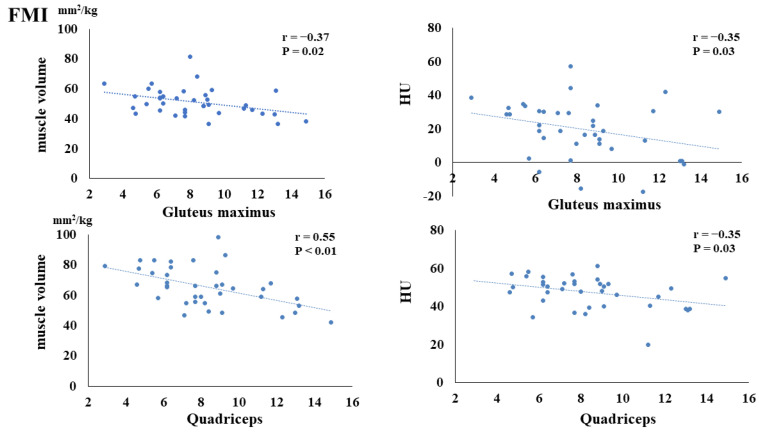
Correlation between preoperative fat mass index (FMI) and postoperative muscle volume and fatty degeneration.

**Figure 4:**
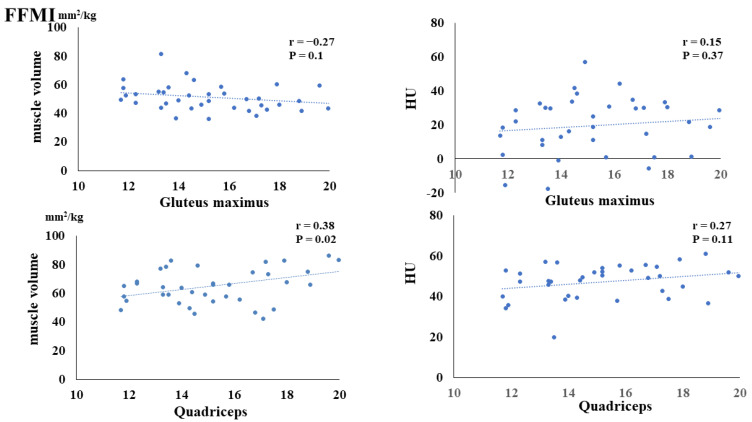
Correlation between preoperative fat-free mass index (FFMI) and postoperative muscle volume and fatty degeneration.

**Figure 5:**
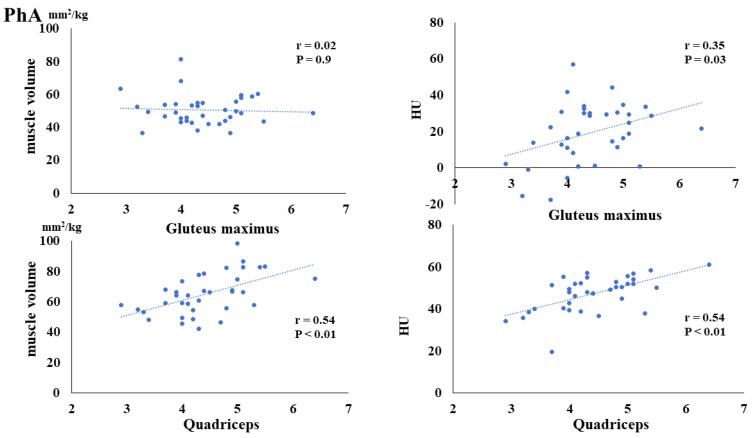
Correlation between preoperative phase angle (PhA) and postoperative muscle volume and fatty degeneration.

The preoperative FMI negatively correlated with the postoperative muscle volume of the gluteus maximus (*p* = 0.02) and quadriceps (*p* < 0.01). Additionally, the preoperative FMI negatively correlated with the HUs of the gluteus maximus (*p* = 0.03) and quadriceps (*p* = 0.03) (Figure 3). The preoperative FFMI positively correlated with postoperative quadriceps muscle mass (*p* = 0.02) (Figure 4). The preoperative PhA positively correlated with the postoperative muscle volume of the quadriceps (*p* < 0.01) and HUs of the gluteus maximus (*p* = 0.03) and quadriceps (*p* < 0.01) (Figure 5).

The intra- and inter-observer agreements regarding muscle volumes were 0.995 (95% CI, 0.992–0.997) and 0.985 (95% CI, 0.958–0.993), respectively, while those for the HU values were 0.996 (95% CI, 0.994–0.998) and 0.988 (95% CI, 0.973–0.994), respectively.

The preoperative FMI was significantly and negatively correlated with the postoperative muscle volume of the gluteus maximus and quadriceps and postoperative Hounsfield Units (HUs) of the gluteus maximus and quadriceps.

The preoperative FFMI was significantly and positively correlated with the postoperative muscle volume of the quadriceps. The preoperative FFMI was not significantly correlated with the postoperative Hounsfield Units (HUs) of the gluteus maximus or quadriceps.

The preoperative PhA was significantly and positively correlated with the postoperative muscle volume of the quadriceps and postoperative Hounsfield Units (HUs) of the gluteus maximus and quadriceps.

## 4. Discussion

Some previous studies evaluated the muscle volume around the hip after THA using CT and reported that the muscle volume was increased after THA [17,18,22,24]. Our results also found significantly increased muscle volume after THA in the gluteus maximus and quadriceps. This may be because the alleviation of hip pain leads to the improvement of daily activities, thus accelerating the increase in muscle volume. Conversely, in this study, the HUs of the gluteus maximus and quadriceps did not change from pre- to postoperatively. Our surgical approach was intermuscular invasion between the gluteus medius and tensor fasciae latae, which prevented injury to the posterior muscles and quadriceps as well as the adipose degeneration of the muscles. This study only assessed the gluteus maximus and quadriceps, which are essential for posture retention and gait, and excluded other hip muscles, such as the gluteus medius, tensor fasciae latae, inter obturator, and external obturator, which could have been injured by the surgical approach.

Although CT can evaluate muscle volume and adipose degeneration, the examination costs and radiation exposure are concerns, and CT can only assess muscle volume planarly. BIA has been used for evaluating metabolic disease and obesity and can assess body composition safely and simultaneously by taking advantage of microcurrent electricity [27,43]. BMI also has been used as an indicator for assessing metabolic disease. However, metabolic health and abnormal obesity cannot be distinguished because the FMI and FFMI cannot be distinguished based on BMI [44]. Merchant et al. reported that a higher FMI was associated with a higher rate of sarcopenia, and there was a possibility that the FFMI and FMI were more useful for predicting the functional outcome in prefrail patients than the BMI [44]. The preoperative FMI was found to correlate both with muscle volume and fatty degeneration after THA in this study. Conversely, the preoperative FFMI was only associated with the muscle volume of the quadriceps. From these results, a detailed assessment of body composition parameters may be important to assess postoperative muscle volume and degeneration. Additionally, the preoperative FMI may be a better predictor of muscle volume and fatty degeneration around the hip after THA than the FFMI.

The PhA is affected by nutritional status [45] and several health indicators [46] and is deeply related to muscle mass [35] and muscle quality [47]. Lower PhA values indicate an increased quantity of extra water and decreased muscle mass [48,49]. The PhA has also been associated with osteoarthritis severity [50], functional ability [34,44,51,52], and Barthel’s index [53]. It is reported that the PhA was associated with muscle strength [54] and quadriceps strength [50]. Therefore, there is a possibility that the PhA might be a useful predictor for screening physical function [55]. In this study, the preoperative PhA correlated with the postoperative HUs, both of the gluteus maximus and quadriceps and muscle volume of the quadriceps. These results also indicate that the preoperative PhA may be a useful prognostic tool for evaluating postoperative muscle volume and fatty degeneration around the hip. Preoperative body composition analysis was not routinely assessed before THA. From our study, body composition outcomes, such as the FMI, FFMI, and PhA, were correlated with postoperative muscle volume and degeneration. Especially, the preoperative FMI was negatively correlated and the preoperative PhA was positively correlated with postoperative muscle volume and degeneration. Therefore, preoperative interventions to decrease the FMI (maintaining an ideal weight) and increase the FFMI (maintaining and increasing skeletal muscle mass) and PhA (improving the nutritional balance) may have a positive impact on increasing muscle volume and preventing the fatty degeneration of muscles.

There were some limitations to this study. First, this study was a retrospective study and the sample size was small. However, to the best of our knowledge, this study is the first to assess body composition before and after THA. Thus, we believe that this study provides new insight into the prediction of skeletal muscle volume and adipose degeneration after THA. Furthermore, a power analysis was performed to ensure that a sufficient sample size was obtained to detect significant differences. However, patients in this study were mixed sex, which might have affected the results. Thus, in the future, we plan to include more patients and evaluate body composition separately for males and females. Second, the CT evaluation was performed manually, which may reduce inter- or intra-rater reliability; however, reliability was demonstrated to be high. The manual measurement of muscle volume and degeneration is frequently performed; nonetheless, the possibility of bias was not eliminated. Thus, measuring muscle volume and degeneration using artificial intelligence will be planned in the next study. Third, this study performed a simple correlation test, and regression analysis is preferred to identify the risk factors for postoperative muscle volume and degeneration. However, we could not perform a regression analysis owing to the insufficient sample size. Regardless, this is the first report to evaluate the correlation between preoperative body composition and postoperative muscle volume and degeneration. We believe this study will make a valuable contribution not only to the orthopedic field, but also to the nutrition field. Fourth, our patients had multiple diagnoses, which may have influenced the muscle volume and adipose degeneration after THA. Third, although it has been reported that the PhA varies dependent on the BMI, the limited sample size restricted BMI variance in this study. Westphal et al. reported that the PhA tended to increase when the BMI was <35 kg/m^2^ and decrease when the BMI was >35 kg/m^2^ [36]. The PhA has also been inversely associated with percent body fat [56] and the degree of obesity [57]. However, there were no patients whose BMI exceeded 35 kg/m^2^, and a significant correlation between the preoperative FMI and PhA was not seen in this study. Fourth, postoperative BIA analysis can be affected by THA because BIA is based on the measurement of the impedance of body tissues to an applied electrical current of low intensity. Although the electrical resistance of fat tissue is very high, the resistances of muscle tissue and metal are low. Thus, postoperative muscle mass may be overestimated. However, another study reported that postoperative electrical resistance was not significantly different compared to preoperative resistance [58]. Therefore, we believe that postoperative BIA analysis is reasonable.

## 5. Conclusions

Preoperative body composition affects postoperative muscle mass after THA and the adipose degeneration of the affected lower limb. Preoperative body composition may be useful for predicting postoperative muscle mass and adipose degeneration.

## Figures and Tables

**Table 1:** Demographic data.

Characteristic	Patients (*n* = 38)
Age (years)	61.6 ± 16.0
Sex (male:female)	15:23
BMI (kg/m^2^)	23.8 ± 5.8
Diagnosis (OA:ION:RA:RDC)	18:17:2:1
Preoperative leg discrepancy (mm)	−9.5 ± 8.2
Postoperative leg discrepancy (mm)	−3.2 ± 8.8
Preoperative offset (mm)	41.0 ± 6.4
Postoperative offset (mm)	40.0 ± 6.4

BMI: Body mass index, OA: Osteoarthritis, ION: Idiopathic osteonecrosis of the femoral head, RA: Rheumatoid arthritis, RDC: Rapidly destructive arthropathy. All values are expressed as mean ± standard deviation.

**Table 2:** Comparisons of pre- and postoperative parameters of muscle strength, body composition, muscle volume, and Hounsfield Units.

	Preoperative	Postoperative	*p* Value
Muscle strength of hip flexion (N/kg)	1.1 ± 0.7	2.2 ± 0.8	<0.01 *
Muscle strength of hip extension (N/kg)	1.7 ± 0.6	2.2 ± 0.4	<0.01 *
Muscle strength of hip abduction (N/kg)	1.3 ± 0.5	1.8 ± 0.4	<0.01 *
FMI (kg/m^2^)	8.3 ± 2.8	8.3 ± 2.5	0.50
FFMI (kg/m^2^)	15.3 ± 2.4	15.5 ± 2.6	<0.01 *
PhA (degrees)	4.4 ± 0.7	4.4 ± 0.7	0.33
Muscle volume of gluteus maximus (mm^2^/kg)	45.9 ± 10.4	51.1 ± 9.2	<0.01 *
HU of gluteus maximus	21.0 ± 13.5	19.7 ± 16.4	0.16
Muscle volume of quadriceps (mm^2^/kg)	65.5 ± 13.1	70.6 ± 13.1	<0.01 *
HU of quadriceps	47.1 ± 8.3	47.4 ± 8.4	0.60

FMI: fat mass index, FFMI: free fat mass index, PhA: phase angle, HU: Hounsfield Units. *: Significant values (*p* < 0.05). All values are expressed as mean value ± standard deviation.

## Data Availability

The datasets used and/or analyzed during the current study are available from the corresponding author upon reasonable request. The data are not publicly available due to ethical restrictions.

## References

[1] Kurtz S., Ong K., Lau E., Mowat F., Halpern M. (2007). Projections of primary and revision hip and knee arthroplasty in the United States from 2005 to 2030. J. Bone Jt. Surg. Am..

[2] Majewski M., Bischoff-Ferrari H.A., Grüneberg C., Dick W., Allum J.H. (2005). Improvements in balance after total hip replacement. J. Bone Jt. Surg. Br..

[3] Lugade V., Klausmeier V., Jewett B., Collis D., Chou L.S. (2008). Short-term recovery of balance control after total hip arthroplasty. Clin. Orthop. Relat. Res..

[4] Rasch A., Dalén N., Berg H.E. (2010). Muscle strength, gait, and balance in 20 patients with hip osteoarthritis followed for 2 years after THA. Acta Orthop..

[5] Judd D.L., Dennis D.A., Thomas A.C., Wolfe P., Dayton M.R., Stevens-Lapsley J.E. (2014). Muscle strength and functional recovery during the first year after THA. Clin. Orthop. Relat. Res..

[6] Denison H.J., Cooper C., Sayer A.A., Robinson S.M. (2015). Prevention and optimal management of sarcopenia: A review of combined exercise and nutrition interventions to improve muscle outcomes in older people. Clin. Interv. Aging.

[7] Fukunaga T., Miyatani M., Tachi M., Kouzaki M., Kawakami Y., Kanehira H. (2001). Muscle volume is a major determinant of joint torque in humans. Acta Physiol. Scand..

[8] Baxter J.R., Piazza S.J. (2014). Plantar flexor moment arm and muscle volume predict torque-generating capacity in young men. J. Appl. Physiol..

[9] Westcott W.L. (2012). Resistance training is medicine: Effects of strength training on health. Curr. Sports Med. Rep..

[10] Mcleod J.C., Stokes T., Phillips S.M. (2019). Resistance exercise training as a primary countermeasure to age-related chronic disease. Front. Physiol..

[11] Tyrovolas S., Panagiotakos D., Georgouspoulou E., Chrysohoou C., Tousoulis D., Haro J.M., Pitsavos C. (2020). Skeletal muscle mass in relation to 10 year cardiovascular disease incidence among middle aged and older adults: The ATTICA study. J. Epidemiol. Commun. Health.

[12] Ferrero E., Skalli W., Lafage V., Maillot C., Carlier R., Feydy A., Felter A., Khalifé M., Guigui P. (2020). Relationships between radiographic parameters and spinopelvic muscles in adult spinal deformity patients. Eur. Spine J..

[13] Bao H., Moal B., Vira S., Bronsard N., Amabile C., Errico T., Schwab F., Skalli W., Dubousset J., Lafage V. (2020). Spino-femoral muscles affect sagittal alignment and compensatory recruitment: A new look into soft tissues in adult spinal deformity. Eur. Spine.

[14] Manabe T., Ogawa C., Takuma M., Nakahara M., Oura K., Tadokoro T., Fujita K., Tani J., Shibatoge M., Morishita A. (2023). Usefulness of the measurement of psoas muscle volume for sarcopenia diagnosis in patients with liver disease. Diagnostics.

[15] Nankaku M., Tsuboyama T., Akiyama H., Kakinoki R., Fujita Y., Nishimura J., Yoshioka Y., Kawai H., Matsuda S. (2013). Preoperative prediction of ambulatory status at 6 months after total hip arthroplasty. Phys. Ther..

[16] Holstege M.S., Lindeboom R., Lucas C. (2011). Preoperative quadriceps strength as a predictor for short-term functional outcome after total hip replacement. Arch. Phys. Med. Rehabil..

[17] Uemura K., Takao M., Sakai T., Nishii T., Sugano N. (2016). Volume increases of the gluteus maximus, gluteus medius, and thigh muscles after hip arthroplasty. J. Arthroplast..

[18] Ukai T., Ebihara G., Omura H., Watanabe M. (2021). Evaluation of muscle volume and degeneration after total hip arthroplasty: A comparison of the posterolateral approach and the anterolateral supine approach. J. Orthop. Surg. Res..

[19] Homma D., Minato I., Imai N., Miyasaka D., Sakai Y., Horigome Y., Suzuki H., Dohmae Y., Endo N. (2023). Relationship between the hip abductor muscles and abduction strength in patients with hip osteoarthritis. Acta Med. Okayama.

[20] Rasch A., Byström A.H., Dalen N., Berg H.E. (2007). Reduced muscle radiological density, cross-sectional area, and strength of major hip and knee muscles in 22 patients with hip osteoarthritis. Acta Orthop..

[21] Liu R., Wen X., Tong Z., Wang K., Wang C. (2012). Changes of gluteus medius muscle in the adult patients with unilateral developmental dysplasia of the hip. BMC Musculoskelet. Disord..

[22] Rasch A., Byström A.H., Dalén N., Martinez-Carranza N., Berg H.E. (2009). Persisting muscle atrophy two years after replacement of the hip. J. Bone Jt. Surg. Br..

[23] Grimaldi A., Richardson C., Stanton W., Durbridge G., Donnelly W., Hides J. (2009). The association between degenerative hip joint pathology and size of the gluteus medius, gluteus minimus and piriformis muscles. Man. Ther..

[24] Ogawa T., Takao M., Otake Y., Yokota F., Hamada H., Sakai T., Sato Y., Sugano N. (2020). Validation study of the CT-based cross-sectional evaluation of muscular atrophy and fatty degeneration around the pelvis and the femur. J. Orthop. Sci..

[25] Milanović Z., Pantelić S., Trajković N., Sporiš G., Kostić R., James N. (2013). Age-related decrease in physical activity and functional fitness among elderly men and women. Clin. Interv. Aging.

[26] Bianchi A.J., Guépet-Sordet H., Manckoundia P. (2015). Changes in olfaction during ageing and in certain neurodegenerative diseases: Up-to-date. Rev. Med. Interne.

[27] Panel N.O. (1998). On the identification, evaluation, and treatment of overweight and obesity in adults. Clinical guidelines on the identification, evaluation, and treatment of overweight and obesity in adults—The evidence report. Obes. Res..

[28] Skrzypek M., Szponar B., Drop B., Panasiuk L., Malm M. (2020). Anthropometric, body composition and behavioural predictors of bioelectrical impedance phase angle in Polish young adults—Preliminary results. Ann. Agric. Environ. Med..

[29] Matthews L., Bates A., Wootton S.A., Levett D. (2021). The use of bioelectrical impedance analysis to predict post-operative complications in adult patients having surgery for cancer: A systematic review. Clin. Nutr..

[30] Pfeifer M., Begerow B., Minne H.W., Schlotthauer T., Pospeschill M., Scholz M., Lazarescu A., Pollähne W. (2001). Vitamin D status, trunk muscle strength, body sway, falls, and fractures among 237 postmenopausal women with osteoporosis. Exp. Clin. Endocrinol. Diabetes.

[31] Sinaki M. (2012). Exercise for patients with osteoporosis: Management of vertebral compression fractures and trunk strengthening for fall prevention. PM R.

[32] Lukaski H.C., Kyle U.G., Kondrup J. (2017). Assessment of adult malnutrition and prognosis with bioelectrical impedance analysis: Phase angle and impedance ratio. Curr. Opin. Clin. Nutr. Metab. Care.

[33] Matias C.N., Monteiro C.P., Santos D.A., Martins F., Silva A.M., Laires M.J., Sardinha L.B. (2015). Magnesium and Phase Angle: A prognostic tool for monitoring cellular integrity in judo athletes. Magnes. Res..

[34] Norman K., Stobäus N., Pirlich M., Bosy-Westphal A. (2012). Bioelectrical phase angle and impedance vector analysis-clinical relevance and applicability of impedance parameters. Clin. Nutr..

[35] Kilic M.K., Kizilarslanoglu M.C., Arik G., Bolayir B., Kara O., Varan H.D., Sumer F., Kuyumcu M.E., Halil M., Ulger Z. (2017). Association of bioelectrical impedance analysis-derived phase angle and sarcopenia in older adults. Nutr. Clin. Pract..

[36] Bosy-Westphal A., Danielzik S., Dörhöfer R.P., Later W., Wiese S., Müller M.J. (2006). Phase angle from bioelectrical impedance analysis: Population reference values by age, sex, and body mass index. JPEN J. Parenter. Enter. Nutr..

[37] Nagano M., Suita S., Yamanouchi T. (2000). The validity of bioelectrical impedance phase angle for nutritional assessment in children. J. Pediatr. Surg..

[38] Pupim L.B., Kent P., Ikizler T.A. (1999). Bioelectrical impedance analysis in dialysis patients. Min. Electrolyte Metab..

[39] Marini E., Campa F., Buffa R., Stagi S., Matias C.N., Toselli S., Sardinha L.B., Silva A.M. (2020). Phase angle and bioelectrical impedance vector analysis in the evaluation of body composition in athletes. Clin. Nutr..

[40] Selberg O., Selberg D. (2002). Norms and correlates of bioimpedance phase angle in healthy human subjects, hospitalized patients, and patients with liver cirrhosis. Eur. J. Appl. Physiol..

[41] Kyle U.G., Soundar E.P., Genton L., Pichard C. (2012). Can phase angle determined by bioelectrical impedance analysis assess nutritional risk? A comparison between healthy and hospitalized subjects. Clin. Nutr..

[42] Chen J., Lu K., Chen H., Hu N., Chen J., Liang X., Qin J., Huang W. (2020). Trunk skeletal muscle mass and phase angle measured by bioelectrical impedance analysis are associated with the chance of femoral neck fracture in very elderly people. Clin. Interv. Aging.

[43] Day K., Kwok A., Evans A., Mata F., Verdejo-Garcia A., Hart K., Ward L.C., Truby H. (2018). Comparison of a bioelectrical impedance device against the reference method dual energy X-ray absorptiometry and anthropometry for the evaluation of body composition in adults. Nutrients.

[44] Merchant R.A., Seetharaman S., Au L., Wong M.W.K., Wong B.L.L., Tan L.F., Chen M.Z., Ng S.E., Soong J.T.Y., Hui R.J.Y. (2021). Relationship of fat mass index and fat free mass index with body mass index and association with function, cognition and sarcopenia in pre-frail older adults. Front. Endocrinol..

[45] Kyle U.G., Genon L., Karsegard V.L., Raguso C.A., Dupertuis Y.M., Pichard C. (2004). Percentile (10, 25, 75 and 90th) for phase angle (PhA), determined by bioelectrical impedance analysis (BIA) in 2740 healthy adults aged 20–75 yr. Clin. Nutr..

[46] Tomeleri C.M., Cavaglieri C.R., de Souza M.F., Cavalcante E.F., Antunes M., Nabbuco H.C.G., Venturini D., Barbosa D.S., Silva A.M., Cyrino E.S. (2018). Phase angle is related with inflammatory and oxidative stress biomarkers in older women. Exp. Gerontol..

[47] Nunes J.P., Ribeiro A.S., Silva A.M., Schoenfeld B.J., dos Santos L., Cunha P.M., Nascimento M.A., Tomeleri C.M., Nabuco H.C.G., Antunes M. (2019). Improvements in phase angle are related with muscle quality index after resistance training in older women. J. Aging Phys. Act..

[48] Souza M.F., Tomeleri C.M., Ribeiro A.S., Schoenfeld B.J., Silva A.M., Sardinha L.B., Cyrino E.S. (2017). Effect of resistance training on phase angle in older women: A randomized controlled trial. Scand. J. Med. Sci. Sports.

[49] Ten Haaf D.S.M., Nuijten M.A.H., Maessen M.F.H., Horstman A.M.H., Eijsvogels T.M.H., Hopman M.T.E. (2018). Effects of protein supplementation on lean body mass, muscle strength, and physical performance in nonfrail community-dwelling older adults: A systematic review and meta-analysis. Am. J. Clin. Nutr..

[50] Wada O., Kurita N., Yamada M., Mizuno K. (2020). Structural severity, phase angle, and quadriceps strength among patients with knee osteoarthritis: The SPSS-OK study. Clin. Rheumatol..

[51] Gunn S.M., Halbert J.A., Giles L.C., Stepien J.M., Miller M.D., Crotty M. (2008). Bioelectrical phase angle values in a clinical sample of ambulatory rehabilitation patients. Dyn. Med..

[52] Tanaka S., Ando K., Kobayashi K., Hida T., Seki T., Hamada T., Ito K., Tsushima M., Morozumi M., Machino M. (2019). The decrease in phase angle measured by bioelectrical impedance analysis reflects the increased locomotive syndrome risk in community-dwelling people: The Yakumo study. Mod. Rheumatol..

[53] Norman K., Stobäus N., Zocher D., Bosy-Westphal A., Szramek A., Scheufele R., Smoliner C., Pirlich M. (2010). Cutoff percentiles of bioelectrical phase angle predict functionality, quality of life, and mortality in patients with cancer. Am. J. Clin. Nutr..

[54] Yamada M., Kimura Y., Ishiyama D., Nishio N., Otobe Y., Tanaka T., Ohji S., Koyama S., Sato A., Suzuki M. (2019). Phase angle is a useful indicator for muscle function in older adults. J. Nutr. Health Aging.

[55] Matias C.N., Nunes C.L., Francisco S., Tomeleri C., Cyrino E., Sardinha L., Silva A. (2020). Phase angle predicts physical function in older adults. Arch. Gerontol. Geriatr..

[56] Streb A.R., Hansen F., Gabiatti M.P., Tozetto W.R., Del Duca G.F. (2020). Phase angle associated with different indicators of health-related physical fitness in adults with obesity. Physiol. Behav..

[57] Barrea L., Muscogiuri G., Laudisio D., Di Somma C., Salzano C., Pugliese G., De Alteriis G., Colao A., Savastano S. (2019). Phase Angle: A possible biomarker to quantify inflammation in subjects with obesity and 25(OH)D deficiency. Nutrients.

[58] Ukai T., Watanabe M. (2023). Do metal implants for total hip arthroplasty affect bioelectrical impedance analysis? A retrospective study. BMC Musculoskelet. Disord..

